# Diagnosed with a Rare Cancer: Experiences of Adult Sarcoma Survivors with the Healthcare System—Results from the SURVSARC Study

**DOI:** 10.3390/cancers13040679

**Published:** 2021-02-08

**Authors:** Cas Drabbe, Dirk J. Grünhagen, Winan J. Van Houdt, Pètra M. Braam, Vicky L. M. N. Soomers, Jos A. Van der Hage, Jacco J. De Haan, Kristien B. M. I. Keymeulen, Olga Husson, Winette T. A. Van der Graaf

**Affiliations:** 1 Department of Medical Oncology, Netherlands Cancer Institute, Plesmanlaan 121, 1066 CX Amsterdam, The Netherlands; cas.drabbe@student.ru.nl (C.D.); w.vd.graaf@nki.nl (W.T.A.V.d.G.); 2 Radboud University Medical Center, Geert Grooteplein Zuid 10, 6525 GA Nijmegen, The Netherlands; 3 Department of Surgical Oncology, Erasmus MC Cancer Institute, Erasmus University Medical Center, Doctor Molewaterplein 40, 3015 GD Rotterdam, The Netherlands; d.grunhagen@erasmusmc.nl; 4 Department of Surgical Oncology, Netherlands Cancer Institute, Plesmanlaan 121, 1066 CX Amsterdam, The Netherlands; w.v.houdt@nki.nl; 5 Department of Radiation Oncology, Radboud University Medical Center, Geert Grooteplein Zuid 10, 6525 GA Nijmegen, The Netherlands; p.braam@radboudumc.nl; 6 Department of Medical Oncology, Radboud University Medical Center, Geert Grooteplein Zuid 10, 6525 GA Nijmegen, The Netherlands; vicky.soomers@radboudumc.nl; 7 Department of Surgical Oncology, Leiden University Medical Center, Albinusdreef 2, 2300 RC Leiden, The Netherlands; j.a.van_der_hage@lumc.nl; 8 Department of Medical Oncology, University Medical Center Groningen, Hanzeplein 1, 9713 GZ Groningen, The Netherlands; j.j.de.haan@umcg.nl; 9 Department of Surgical Oncology, Maastricht University Medical Center, P. Debyelaan 25, 6229 HX Maastricht, The Netherlands; k.keymeulen@mumc.nl; 10 Division of Clinical Studies, Institute of Cancer Research, 15 Cotswold Road, Sutton, London SM2 5NG, UK; 11 Department of Medical Oncology, Erasmus MC Cancer Institute, Erasmus University Medical Center, Doctor Molewaterplein 40, 3015 GD Rotterdam, The Netherlands

**Keywords:** rare cancer, survivorship, sarcoma, experience with healthcare, satisfaction with care, information needs, age-related, adolescent and young adult, elderly

## Abstract

**Simple Summary:**

Patients with rare cancers face obstacles including delays in diagnosis, inadequate treatments and limited scientific evidence to guide decision making. These obstacles may have a unique impact on their experience with the healthcare system and might be different at various ages. Some aspects of care that shape the experience with the healthcare system include information needs, satisfaction with care and supportive care. Very little is known about these aspects of care, specifically for rare cancer patients. Sarcomas are prime examples of rare cancers and are diagnosed at all ages. In this study, we explored the experience of sarcoma patients (*N* = 1099) with the healthcare system and looked into detail at whether differences in experience existed between age groups. The results of this nationwide study showed that healthcare experiences differ per age group and we identified needs related to the rarity of these tumors, such as improvements concerning (non-)medical guidance and diagnostic intervals.

**Abstract:**

The aim of this study was to explore the experience of rare cancer patients with the healthcare system and examine differences between age groups (adolescents and young adults (AYA, 18–39 years), older adults (OA, 40–69 years) and elderly (≥70 years)). Dutch sarcoma patients, 2–10 years after diagnosis, completed a questionnaire on their experience with the healthcare system, satisfaction with care, information needs, patient and diagnostic intervals (first symptom to first doctor’s visit and first doctor’s visit to diagnosis, respectively) and received supportive care. In total, 1099 patients completed the questionnaire (response rate 58%): 186 AYAs, 748 OAs and 165 elderly. Many survivors experienced insufficient medical and non-medical guidance (32% and 38%), although satisfaction with care was rated good to excellent by 94%. Both patient and diagnostic intervals were >1 month for over half of the participants and information needs were largely met (97%). AYAs had the longest patient and diagnostic intervals, experienced the greatest lack of (non-)medical guidance, had more desire for patient support groups and used supportive care most often. This nationwide study among sarcoma survivors showed that healthcare experiences differ per age group and identified needs related to the rarity of these tumors, such as improvements concerning (non-)medical guidance and diagnostic intervals.

## 1. Introduction

Rare cancers are cancer types with fewer than 6 cases per 100,000 people per year. Despite the rarity of these individual cancer types, the collective burden of this extremely heterogenous group is substantial, with 24% of all cancer diagnoses being of a rare kind. Rare cancers should therefore be recognized as a public health priority [1]. The 5-year overall survival of patients with rare cancers is 48.5% and considerably worse than in patients with more common cancers (63.4%) [2]. These inferior outcomes for rare cancer patients are mainly ascribed to specific obstacles rare cancer patients face, including delay in diagnosis, absence of expert care, inadequate treatments and the lack of scientific evidence to guide decision making [3,4].

The obstacles rare cancer patients face may have a unique impact on their experience with the healthcare system. The limited scientific evidence to guide decision making, delayed diagnostic pathways and harder to find expert care may lead to a different experience with the healthcare system compared with common cancer patients. There are many aspects of care studied in cancer in general or in common cancers that shape the experience with the healthcare system, including information needs, satisfaction with care and supportive care [5,6,7,8]. Although the healthcare system for rare cancer patients has been researched from a policy perspective [9,10], very little is known about the perspective of rare cancer patients on their healthcare system experience.

Sarcomas are a typical example of rare cancers. As a group, they are extremely heterogenous, not only because of the more than 70 histological subtypes, but also in terms of anatomical location and biological behavior [11,12]. Additionally, sarcomas possess a very diverse nature across the age spectrum as the incidence of different histological sarcoma subtypes differs largely by age. Sarcomas represent 11% of pediatric (<15 years) cancers [13], 11% of adolescent and young adult (AYA, 15–29 years) cancers [14] and approximately 1% of adult (≥18 years) cancers [15]. Within the Dutch healthcare system, the centralization of care for adult sarcoma patients increasingly takes place, however this is not always the case. Decentralized care may lead to a long route to final and accurate diagnosis and a lack of expert care, which in turn leads to a worse experience with the healthcare system [16].

In addition to the diverse nature of sarcomas across the age spectrum, the challenges that cancer patients face vary greatly at different ages. AYA patients are confronted with the diagnosis at an emotionally, cognitively and socially challenging time in their lives, which interferes with the achievement of common developmental milestones [17]. Elderly patients face distinct challenges, such as comorbidity, that may hamper optimal treatment [18]. A recent study on the age-related experience of sarcoma survivors reported that AYA patients are more vulnerable to incorrect diagnosis, have a high burden of treatment-related side effects and post-treatment psychological concerns, whereas elderly survivors were less likely to be referred to rehabilitation services and reported less trial participation [19]. The experience of rare cancer patients with the healthcare system might therefore be age-dependent. For instance, younger patients tend to be more assertive, which is a favorable trait when expert care is not always readily available. On the other hand, younger patients often lack experience with healthcare systems, while older patients generally have more experience which may help them to navigate.

To our knowledge, there has not been any research conducted that comprehensively discusses the experience with the healthcare system from a rare cancer patients’ perspective. We hypothesize that there are age-related aspects of relevance in the experience with the healthcare system for rare cancer patients. Therefore, we explored the results of the Survivorship (SURVSARC) study, a cross-sectional questionnaire study among Dutch adult sarcoma survivors, in which we studied multiple aspects of the experience of rare cancer patients with the healthcare system.

## 2. Materials and Methods

### 2.1. Study Design and Participants

**Figure 1:**
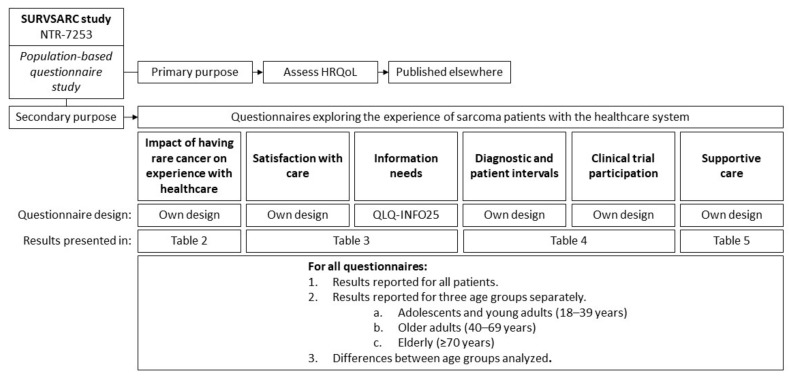
Overview of the design and measures from the SURVSARC study used in this manuscript. HRQoL: Health-related quality of life. QLQ-INFO25: European Organization for Research and Treatment of Cancer Quality of Life Group information questionnaire.

The SURVSARC study is a nationwide cross-sectional questionnaire study among adult (≥18 years) sarcoma survivors, registered in the Netherlands Cancer Registry (NCR). The primary purpose of the SURVSARC study was to assess health-related quality of life (HRQoL). The results presented here are secondary analyses, consisting of six questionnaires from the SURVSARC study that are relevant to the experience with the healthcare system (Figure 1). Sarcoma survivors diagnosed between 1 January 2008 and 31 December 2016 at one of the six participating sarcoma expertise centers (Radboudumc Nijmegen, Antoni van Leeuwenhoek/The Netherlands Cancer Institute Amsterdam, University Medical Center Groningen, Leiden University Medical Center, Erasmus MC Cancer Institute Rotterdam, Maastricht University Medical Center) were eligible. Exclusion criteria were cognitive impairment and physical condition too poor to participate. Survivors with desmoid fibromatosis, grade I chondrosarcoma, gastrointestinal stromal tumors, atypical lipomatous tumors or giant-cell tumors were also excluded considering the indolent clinical behavior and less aggressive treatment strategies for these tumors.

### 2.2. Recruitment and Data Collection

Eligible sarcoma survivors received a letter from their (former) treating healthcare professional, describing the purpose of the study. If informed consent was obtained, participants were able to complete the questionnaire. The NCR contains data on patient and tumor characteristics of all newly diagnosed cancer patients in The Netherlands. Completion of the questionnaire was conducted between October 2018 and June 2019 within the PROFILES (Patient Reported Outcomes Following Initial treatment and Long-term Evaluation of Survivorship) data management system, patients were therefore 2–10 years after diagnosis [20].

### 2.3. Study Measures

Sociodemographic and tumor characteristics, including sex, date of birth, date of diagnosis, histological subtypes, tumor grade, stage at diagnosis and localization of the primary tumor, were obtained from the NCR. The six questionnaires from the SURVSARC study that are relevant to the experience of rare cancer patients with the healthcare system evaluate the impact of having a rare cancer on the experience with healthcare, satisfaction with care, information needs, diagnostic and patient intervals, clinical trial participation and supportive care (Figure 1). All are described in detail below. In order to investigate age-related differences of rare cancer patients’ experience with the healthcare system, participants were divided into three age categories according to their age at diagnosis: adolescents and young adults (AYA, 18–39 years), older adults (OA, 40–69 years) and elderly survivors (≥70 years). Marital status, educational level and employment status were self-reported by the participants.

#### 2.3.1. Impact of Having a Rare Cancer on the Experience with the Healthcare System

A questionnaire assessing the impact of having a rare cancer on the experience with the healthcare system was designed by the study team. This was done in collaboration with sarcoma survivors affiliated with patient support groups in order to learn what issues affected their experience with the healthcare system and incorporate this into the questionnaire. It contains questions exploring domains such as perceived knowledge of healthcare professionals, medical guidance, loneliness and media attention for rare cancers. A four-point Likert scale was used for all twenty questions: totally disagree, somewhat agree, strongly agree and totally agree. For statistical analyses, the answering scale was dichotomized for better power: totally disagree was adjusted into disagree, and somewhat agree, strongly agree and totally agree were grouped into agree. Questions that were formulated in present tense applied to the time of filling out the questionnaire. Some questions were specifically formulated in the past tense, in which case they applied to the diagnostic trajectory.

#### 2.3.2. Satisfaction with Care and Information Needs

Satisfaction with care was assessed by one question, with five answering options (bad, reasonable, good, very good, excellent). Three questions from the EORTC QLQ-INFO25 (European Organization for Research and Treatment of Cancer Quality of Life Group information questionnaire) focused on survivors’ satisfaction with the received information during the diagnostic and therapeutic process and whether more or less information would have been desired by the survivor [21]. Consecutively, survivors could indicate on what subject more or less information was desired. More than one subject could be brought forward by one sarcoma survivor. The subjects on which survivors desired more or less information were categorized by the research team.

#### 2.3.3. Diagnostic and Patient Intervals, Clinical Trial Participation and Supportive Care

The time between first experiencing symptoms and consulting any doctor is considered patient interval, and the time between the first doctor visit and receiving the diagnosis is considered diagnostic interval. Time intervals were patient-reported and categorized into shorter or longer than one month, based on existing literature [22]. The Dutch Foundation for multidisciplinary oncological collaboration (SONCOS) guidelines deems a period of four weeks between general practitioner (GP) referral and histological tumor diagnosis acceptable [23].

The possibility of clinical trial participation was also assessed in the questionnaire. Survivors were asked if they received supportive care, which was defined as care given for the prevention and control of complications, side effects and symptoms in order to improve quality of life (e.g., by a physiotherapist, nurse, dietician, psychologist, general practitioner) and is additional to the care of their sarcoma specialist [24].

### 2.4. Statistical Analyses

An anonymous comparative analysis between responders and non-responders was conducted by an NCR employee and non-responder data was not shared with the research team. An age-stratified comparative analysis of sociodemographic and tumor characteristics of all responding sarcoma survivors was conducted. Chi square tests and one-way analyses of variance (ANOVA) were used for categorial and continuous variables, respectively.

In the comparative analysis between age groups concerning subjects on which patients desired more information, not all categories were included in the statistical analyses due to low numbers in those excluded categories.

All statistical analyses were performed using SPSS Statistics (IBM Corporation, version 26.0, Armonk, NY, USA) and *p*-values < 0.05 were considered statistically significant.

## 3. Results

### 3.1. Responders versus Non-Responders

In total, 1887 sarcoma survivors were approached, of whom 1099 completed the questionnaire. The response rate in AYAs was 41%, in OAs 66% and in elderly survivors 54%. Of the 788 non-responders, data was missing in the NCR database from two survivors. An age-stratified comparative analysis between the 1099 responders and 786 non-responders was done (Table 1).

### 3.2. Sociodemographic and Tumor Characteristics

Of all 1099 sarcoma survivors, 186 (17%) were AYAs (18–39 years), 748 (68%) OAs (40–69 years) and 165 (15%) were elderly (≥70 years) survivors, based on their age at diagnosis. The median time since diagnosis for all patients was 5.2 years (range, 1.7–11.3). The median age at time of the questionnaire was 37 years for AYAs, 62 years for OAs and 80 years for elderly survivors. Of all survivors, 54% were male, whereas 45% of AYAs were male, and 54% of OAs and 66% of elderly survivors were male (*p* < 0.01). Amongst AYAs, 78% had a partner, as did 83% of OAs and 68% of elderly survivors (*p* < 0.01). AYAs were most often highly educated (52%), followed by OAs (37%) and elderly (21%) age groups (*p* < 0.01). Histological subtypes were divergently distributed across age groups.

### 3.3. Impact of Having a Rare Cancer on the Experience with the Healthcare System

#### 3.3.1. All Ages

The results of the questionnaire are presented in Table 2. The vast majority (86%) of survivors agreed that their general practitioner currently has sufficient knowledge about their cancer to deliver the supportive care they require and provide guidance within the healthcare system. Almost all survivors (99%) agreed that their medical specialist has the knowledge to deliver the required care. Almost half of all survivors (45%) reported that it had taken them much effort in the past to receive the final diagnosis. A second opinion was desired by a large number of survivors during the diagnostic or therapeutic process (28%). Insufficient medical guidance experienced at the moment of questionnaire was reported in 32% and an insufficiency of non-medical guidance, such as psychological support or provision of practical information, by 38%. A desire for contact with fellow survivors was reported by 27% and 41% were familiar with the possibility for contact with fellow survivors. Patient support groups would be appreciated by 33%, and 16% would like to actively participate in one. Almost one-third (29%) find it difficult that there is no attention for their disease in the media or in fundraising.

#### 3.3.2. Age-Related

Several statistically significant differences were observed between the three age groups (Table 2). AYAs were more often understanding towards their general practitioner and other caregivers for not having extensive knowledge about their disease since it is rare (*p* = 0.004). AYAs also reported more frequently that it had taken them much effort in the past to receive the final diagnosis (58%), compared to OAs (43%) and elderly (40%) survivors (*p* = 0.001). Elderly survivors least often reported a desire for a second opinion during their diagnostic or therapeutic process (*p* = 0.022). A lack of non-medical guidance was most frequently reported by AYAs (*p* = 0.003). AYAs indicated most frequently to have a desire for contact with fellow survivors (34%, *p* = 0.003), that they would appreciate patient support groups (43%, *p* = 0.004), and would like to actively participate in one (31%, *p* < 0.001).

### 3.4. Satisfaction with Care and Information Needs

#### 3.4.1. All Ages

The vast majority of survivors rated their satisfaction with the care they received either very good (34%) or excellent (35%) (Table 3). Almost all survivors were satisfied with the amount of information they had received (97%). In total, 240 survivors (23%) desired more information and they collectively mentioned 280 topics on which they would have wanted more information. The subjects that were brought forward were categorized and this resulted in the following categories: clarity on diagnosis (4%), sarcoma (17%), treatment (26%), future perspective (31%), psychosocial impact (4%), practical information (3%), other (8%, e.g., fertility, lifestyle, heredity), scientific developments (5%), and a general comment or just the wish of more information was given by 4% of all survivors. Fourteen survivors would have wanted less information, however the comments made by these survivors were mostly of general nature and no specific topics were raised.

#### 3.4.2. Age-Related

The subjects on which survivors would have wanted more information appeared to be different between age groups (*p* = 0.021) (Table 3). More information on sarcomas in general and the rarity of sarcomas was most often desired by elderly survivors in comparison to AYAs and OAs. AYAs most often desired more information about their future perspective, which was mentioned to a lesser extent in the other age groups. Other subjects that were most often named by AYAs were more information on the psychosocial impact of their cancer and practical information.

### 3.5. Diagnostic and Patient Interval, Clinical Trial Participation, and Supportive Care

#### 3.5.1. All Ages

Patient intervals were >1 month for 60% of all survivors and diagnostic intervals were >1 month for 56% (Table 4). In total, 9% were treated within a trial and for 78%, the possibility of being treated within a trial was never discussed. Supportive care (Table 5) was received by 38% of all survivors, most often from a physiotherapist (29%), general practitioner (16%), or psychologist (12%). In all age groups, supportive care was most often received in the form of physiotherapy.

#### 3.5.2. Age-Related

AYAs had both a statistically significant longer diagnostic interval (63%, *p* = 0.001) and patient interval (68%, *p* = 0.001) compared with OAs and elderly. Elderly survivors received supportive care least often (33%, *p* < 0.001). Survivors of different ages received different forms of supportive care in addition to the care from their sarcoma specialist (*p* = 0.001). AYA survivors visited a psychologist most often and OA and elderly survivors received care from their general practitioner most. Elderly survivors received relatively more pastoral support than younger survivors, whereas younger survivors received relatively more care from fellow survivors.

## 4. Discussion

This nationwide study amongst sarcoma survivors who were treated in sarcoma expertise centers explored the experience of rare cancer patients with the healthcare system. They had a general satisfaction with care and high trust in medical professionals. Information needs were largely met, and supportive care was received by 38%. Patient and diagnostic intervals of >1 month were often observed, and patients often reported that insufficient medical and non-medical guidance was experienced. Firstly, we will discuss the most striking results for all patients, then the age-related results and the limitations of the study, and lastly, we will discuss the practical implications.

Over half (56%) of the rare cancer patients in this study reported diagnostic intervals of >1 month. The primary analysis of the diagnostic intervals of this SURVSARC study has been published previously [25]. Diagnostic intervals in common cancers vary greatly between different cancer types. A study investigating diagnostic intervals of common cancers in the Dutch healthcare system showed that symptomatic breast cancer and melanoma had median diagnostic intervals of 7 and 17 days, respectively. On the other hand, colorectal (54 days), lung (49 days), and prostate cancer (137 days) had much longer median diagnostic intervals [26]. Considering the heterogenous presentation of sarcomas, it is difficult to say whether the diagnostic intervals in this rare cancer patient population are significantly different from common cancer patients. In addition, a recent systematic review on the sarcoma diagnostic interval found no clinically relevant clear cut-off point discriminating between short and long total interval [22]. Concerning the experience with the healthcare system, the results from this study show that patients often report that it had cost them much effort to receive the final diagnosis, meaning that from a patient’s perspective, there is room for improvement. An Australian qualitative study researching the perspective of sarcoma health professionals and patients on prolonged diagnostic intervals identified the need for better diagnostic pathways, the incorporation of sarcoma education in medical courses, and a public health approach to improve health-seeking behavior [27]. A qualitative study on the diagnostic route in sarcoma, including Dutch and English patients, identified aspects that could be improved, such as better awareness of sarcoma amongst healthcare providers and patients, better patient empowerment, and having a lead clinician [28]. Ideally, these results should be verified through quantitative research. Furthermore, research investigating clinical characteristics associated with prolonged prognostic intervals may shed light on aspects of the diagnostic route that can be improved.

Existing literature on rare cancer patients’ experiences with the healthcare system is very limited. Research on rare cancers largely concerns the delays in diagnosis and the uncertainty in decision making due to the scarcity of scientific evidence [3,4,29,30], which is in line with what the rare cancer patients in this study experienced. Literature on the experience with healthcare of patients with a rare disease, not limited to rare cancer, is more widely available. Research shows that patients with serious diseases prefer a more physician-dominated process [31]. However, patients with rare diseases are often more likely to become an expert of their own disease due to a dearth of scientific evidence to guide decision making [32]. Therefore, rare cancer patients may take on a more assertive role during their treatment process, whereas a physician-dominated process is preferred. This could explain the insufficient medical guidance experienced by this study population. Although this is one possible explanation, future research should be undertaken to investigate in more detail why so many of these rare cancer patients experience insufficient (non-)medical guidance. For instance, a qualitative study amongst rare cancer patients could be undertaken to specifically explore what is experienced as insufficient medical guidance during their diagnostic and treatment process and what can be improved for better (non-)medical guidance.

Several age-related issues were observed. In comparison with OAs and elderly, AYAs had more need to connect with fellow sufferers, experienced less non-medical guidance, had the longest patient and diagnostic intervals, and used supportive care most often. By itself, it is rare to be diagnosed with cancer during adolescence or young adulthood and therefore some of the results from this study will be applicable to AYA patients with common cancers as well [12]. Delays in diagnosis in AYA patients are common [33], and in this study population, over two thirds of these AYAs experienced both patient and diagnostic intervals of >1 month, which might be attributable to not only the rarity of being diagnosed with cancer at such a young age but also having a rare cancer. Another factor might be that AYA patients were more often diagnosed with bone sarcomas, which are generally harder to diagnose than soft tissue sarcomas. Concerning information needs, our results showed that AYA patients desired more information about their future perspective and psychosocial impact of their cancer more frequently than older survivors.

Elderly sarcoma patients least often indicated that they experienced insufficient (non-)medical guidance, and least frequently had patient and diagnostic intervals > 1 month compared to the younger age groups. These results suggest that elderly sarcoma patients find their way better in the healthcare system (e.g., familiarity with the healthcare system due to comorbidities) in comparison with younger patients and navigate faster. Furthermore, as cancer becomes more prevalent at an older age, both patients and doctors might be more inclined to think towards a possible malignancy and therefore refer faster and more frequently. Elderly patients in this study population did not have much desire for contact with fellow sufferers or a patient support group and least often received supportive care. This is visible in our finding that elderly patients least often indicated that they experienced insufficient non-medical guidance.

Limitations of this study include a possible response bias, since it is unknown whether survivors did not participate due to poor health or an absence of symptoms [34]. Furthermore, the questionnaire on the impact of having a rare cancer used in this study was self-developed and not validated, illustrating the lack of research on this topic. However, most questions are patient-reported experiences and not patient-reported outcomes for which validation is of greater importance. The heterogeneity of sarcoma subtypes and therefore the high variety in treatments has not been incorporated into the analyses, as this is less relevant for the experience with the healthcare system. Notably, these results were obtained from sarcoma survivors exclusively, because we felt that their experience could be exemplary for other patients with rare malignancies and sarcoma patients are generally well-represented across rare cancer advocacy groups. As these results are obtained from Dutch sarcoma survivors exclusively, caution has to be taken when generalizing to other international healthcare systems. Patients included in this study were diagnosed 2–10 years ago, and in that time period, there have not been any major changes in the healthcare system for these patients, but there has been an increase in availability of digital information such as information about their disease, diagnosis, treatment, expert care centers and patient support groups.

Practical implications are most prominently aimed at reducing the high amount of patient and diagnostic intervals of >1 month. Not only is timely recognition of cancer important for survival [35], it is also associated with a better patient experience [22,36] and therefore the importance of shortening diagnostic intervals is once again emphasized. Quantitative research can help in defining which approach is most effective and efficient in reducing diagnostic intervals, which is the first step in tackling this issue. A realistic first step would be to ensure that all basic medical curricula teach the alarm symptoms for rare cancers, which may lead to more awareness in primary healthcare professionals and hopefully faster referrals. The insufficient (non-)medical guidance experienced by many of these rare cancer patients deserves further investigation, as suggested before. For current practice, healthcare professionals treating rare cancer patients can actively discuss this issue with their patients in order to clarify the individual patient’s need and possibly adapt their own role in the process.

## 5. Conclusions

This nationwide study, which explored the experience of rare cancer survivors with the healthcare system, demonstrates long patient and diagnostic intervals and insufficient medical and non-medical guidance. Furthermore, it shows that healthcare experiences differ per age group, with younger patients experiencing both longer diagnostic intervals and more often insufficient non-medical guidance. This emphasizes the need for a better and more age-adjusted guidance of patients with rare cancers, such as sarcomas, both in the diagnostic and the therapeutic trajectory.

## Figures and Tables

**Table 1:** Responders compared to non-responders according to age groups.

Age Groups ^1^	AYA (18–39 Years)		OA (40–69 Years)		Elderly (≥70 Years)	
	Responders *N* = 186	Non- Responders *N* = 263	Responders *N* = 747	Non- Responders *N* = 378	Responders *N* = 164	Non- Responders *N* = 144
Gender						
Female	102 (55)	120 (46)	343 (46)	186 (49)	57 (35) **	74 (51)
Male	84 (45)	143 (54)	404 (54)	192 (51)	107 (65)	70 (49)
Age at time of the study ^2^	36.3 (7.3) **	35.0 (7.1)	62.2 (8.6) **	60.3 (9.0)	81.5 (4.9) **	83.2 (5.7)
Age at time of diagnosis ^2^	30.0 (6.6) **	29.1 (6.6)	56.6 (8.3) **	54.5 (8.7)	76.6 (4.5) **	77.5 (5.5)
Months since diagnosis ^2,3^	75.7 (30.8) **	70.2 (31.8)	67.3 (30.3) **	69.7 (30.6)	58.6 (26.8) **	68.6 (30.5)
Histologic subtype						
Bone sarcoma	74 (39.8)	87 (33.1)	162 (21.7)	65 (17.2)	27 (16.5)	20 (13.9)
Osteosarcoma	29 (15.6)	36 (13.7)	35 (4.7)	14 (3.7)	6 (3.7)	3 (2.1)
Chondrosarcoma	26 (14.0)	23 (8.7)	89 (11.9)	37 (9.8)	15 (9.1)	12 (8.3)
Chordoma	1 (0.5)	6 (2.3)	22 (2.9)	10 (2.7)	6 (3.7)	3 (2.1)
Ewing sarcoma	16 (8.6)	16 (6.1)	12 (1.6)	3 (0.8)	0 (0.0)	2 (1.4)
Other bone sarcomas	2 (1.1)	6 (2.3)	4 (0.5)	1 (0.3)	0 (0.0)	0 (0.0)
Soft tissue sarcoma	112 (60.2)	176 (66.9)	585 (78.3)	313 (82.8)	137 (83.5)	124 (86.1)
Liposarcoma ^4^	25 (13.4)	30 (11.4)	128 (17.1)	62 (16.4)	24 (14.6)	16 (11.1)
Myxofibrosarcoma	4 (2.2)	12 (4.6)	95 (12.7)	51 (13.5)	37 (22.6)	26 (18.1)
DFSP	24 (12.9) **	61 (23.2)	47 (6.3) **	48 (12.7)	3 (1.8)	0 (0.0)
Leiomyosarcoma	9 (4.8)	9 (3.4)	87 (11.7)	53 (14.0)	18 (11.0)	20 (13.9)
Rhabdomyosarcoma	6 (3.2)	6 (2.3)	9 (1.2)	4 (1.1)	0 (0.0)	1 (0.3)
MPNST	11 (5.9)	6 (2.3)	20 (2.7)	16 (4.2)	3 (1.8)	2 (1.4)
Synovial sarcoma	10 (5.4)	17 (6.5)	24 (3.2) *	4 (1.1)	1 (0.6)	4 (2.8)
Vascular sarcoma	2 (1.1)	3 (1.1)	30 (4.0)	12 (3.2)	11 (6.7)	12 (8.3)
Other soft tissue sarcoma	21 (11.2)	32 (12.2)	145 (19.4)	63 (16.7)	40 (24.4)	43 (29.9)
Localization						
Head and Neck	15 (8.1)	21 (8.0)	48 (6.4)	17 (4.5)	6 (3.7)	11 (7.6)
Thoracic	18 (9.7) **	7 (2.7)	51 (6.8)	27 (7.1)	12 (7.3)	8 (5.6)
Abdominal (no urogenital) ^5^	10 (5.4)	6 (2.3)	75 (10.0)	27 (7.1)	17 (10.4)	13 (9.0)
Gynecological	1 (0.5)	7 (2.7)	16 (2.1)	17 (4.5)	2 (1.2)	5 (3.5)
Urological	0 (0.0)	1 (0.4)	10 (1.3)	8 (2.1)	1 (0.6)	2 (1.4)
Extremities ^6^	85 (45.7)	133 (50.1)	356 (47.7) *	155 (41.0)	73 (44.5)	69 (47.9)
Breast	2 (1.1)	3 (1.1)	16 (2.1)	8 (2.1)	6 (3.7)	7 (4.9)
Pelvis	12 (6.5)	11 (4.2)	58 (7.8)	29 (7.7)	13 (7.9)	10 (6.9)
Skin	26 (14.0) **	63 (24.0)	71 (9.5) **	67 (17.7)	24 (14.6)	15 (10.4)
Other localization	17 (9.1)	11 (4.1)	46 (6.2)	23 (6.1)	10 (6.1)	4 (2.8)
Stage of disease						
Stage IA	38 (20.4)	48 (18.3)	135 (18.1)	67 (17.7)	31 (18.9)	26 (18.1)
Stage IB	33 (17.7)	47 (17.9)	147 (19.7)	84 (22.2)	29 (17.7)	26 (18.1)
Stage IIA	39 (21.0) **	30 (11.4)	143 (19.1)	66 (17.5)	40 (24.4)	24 (16.7)
Stage IIB	15 (8.1)	22 (8.4)	65 (8.7) **	15 (4.0)	13 (7.9)	19 (13.2)
Stage III	10 (5.4)	11 (4.2)	98 (13.1)	35 (9.3)	26 (15.9)	21 (14.6)
Stage IV	6 (3.2)	5 (1.9)	15 (2.0)	8 (2.1)	2 (1.2)	1 (0.7)
Stage IVA	2 (1.1)	4 (1.5)	2 (0)	0	0	1 (0.7)
Stage IVB	2 (1.1)	5 (1.5)	1 (0)	0	0	0 (0)
Unknown	41 (22.0) **	91 (34.6)	141 (18.9) **	103 (27.2)	23 (14.0)	26 (18.1)

This anonymous comparative analysis was conducted by an employee from the Netherlands Cancer Registry. ^1^ Age groups based on age at diagnosis. ^2^ Mean ± standard deviation (SD). ^3^ Months between diagnosis and filling out the questionnaire. ^4^ No statistically significant difference between subtypes of liposarcoma, including pleomorphic liposarcoma, myxoid liposarcoma, undifferentiated liposarcoma. ^5^ No statistically significant difference between intra- and retro-peritoneal tumors. ^6^ No statistically significant difference between tumors in upper and lower extremities. * *p*-value < 0.05. ** *p*-value <0.01. AYA: Adolescents and young adults. OA: Older adults. DFSP: Dermatofibrosarcoma protuberans, MPNST: Malignant peripheral nerve sheath tumor.

**Table 2:** The impact of having a rare cancer on the experience with the healthcare system.

Impact of Having Rare Cancer on Experience with Healthcare System	Total *N =* 1099	AYA (18–39) *N* = 186	OA (40–69) *N =* 748	Elderly (≥70) *N =* 165	*p*-Value
My general practitioner has sufficient knowledge about my disease to deliver the care I need.					0.189
Agree	893 (85.6)	140 (81.4)	621 (86.1)	132 (88.0)	
Disagree	150 (14.4)	32 (18.6)	100 (13.9)	18 (12.0)	
Missing	56	14	27	15	
My medical specialist has sufficient knowledge about my disease to deliver the care I need.					0.030 ^1^
Agree	1036 (99.2)	173 (100)	716 (99.4)	147 (97.4)	
Disagree	8 (0.8)	0 (0.0)	4 (0.6)	4 (2.6)	
Missing	55	13	28	14	
I desire more information on where to find good doctors and hospitals for my disease.					0.683
Agree	329 (31.6)	52 (30.1)	233 (32.4)	44 (29.3)	
Disagree	713 (68.4)	121 (69.9)	486 (67.6)	106 (70.7)	
Missing	57	13	29	15	
I understand that my general practitioner or other caregivers do not know a lot about my disease, because it is rare.					0.004
Agree	846 (81.5)	154 (89.0)	582 (81.1)	110 (74.8)	
Disagree	192 (18.5)	19 (11.0)	136 (18.9)	37 (25.2)	
Missing	61	13	30	18	
Since there is little information available, I feel/felt alone with my disease.					0.282
Agree	411 (39.4)	76 (43.9)	282 (39.2)	53 (35.3)	
Disagree	631 (60.6)	97 (56.1)	437 (60.8)	97 (64.7)	
Missing	57	13	29	15	
Because I know more about my disease than my general practitioner, he/she gives me more responsibility concerning decisions.					0.769
Agree	261 (25.2)	41 (23.7)	180 (25.1)	40 (27.2)	
Disagree	776 (74.8)	132 (76.3)	537 (74.9)	107 (72.8)	
Missing	62	13	31	18	
I find it difficult always having to explain what disease I have.					0.675
Agree	332 (31.9)	60 (34.7)	224 (31.2)	48 (32.2)	
Disagree	708 (68.1)	113 (65.3)	494 (68.8)	101 (67.8)	
Missing	59	13	30	16	
It has cost me a lot of effort to make sure I received the right diagnosis.					0.001
Agree	471 (45.2)	101 (58.4)	311 (43.3)	59 (39.6)	
Disagree	570 (54.8)	72 (41.6)	408 (56.7)	90 (60.4)	
Missing	58	13	29	16	
Because I have a rare disease, there is a lack of medical guidance.					0.736
Agree	348 (33.4)	62 (35.8)	238 (33.1)	48 (32.0)	
Disagree	693 (66.6)	111 (64.2)	480 (66.9)	102 (68.0)	
Missing	58	13	30	15	
Because I have a rare disease, there is a lack of non-medical guidance, such as psychological support or practical information.					0.003
Agree	391 (37.6)	84 (48.6)	259 (36.0)	48 (32.2)	
Disagree	650 (62.4)	89 (51.4)	460 (64.0)	101 (67.8)	
Missing	58	13	29	16	
It is unclear who co-ordinates my care (who is my treating physician).					0.052
Agree	185 (17.8)	35 (20.2)	115 (16.0)	35 (23.8)	
Disagree	854 (82.2)	138 (79.8)	604 (84.0)	112 (76.2)	
Missing	60	13	29	18	
Most of the time I am informed of treatment options before my treating physician is.					0.896
Agree	81 (7.8)	13 (7.5)	55 (7.6)	13 (8.7)	
Disagree	960 (92.2)	160 (92.5)	664 (92.4)	136 (91.3)	
Missing	58	13	29	16	
I always have to explain to new caregivers what kind of disease I have (had).					0.715
Agree	322 (30.9)	58 (33.5)	218 (30.3)	46 (30.9)	
Disagree	719 (69.1)	115 (66.5)	501 (69.7)	103 (69.1)	
Missing	58	13	29	16	
I have a desire for contact with people who have (had) the same disease as me.					0.003
Agree	276 (26.6)	58 (33.5)	193 (26.9)	25 (16.9)	
Disagree	762 (73.4)	115 (66.5)	524 (73.1)	123 (83.1)	
Missing	61	13	31	17	
There is a possibility for contact with people who have (had) the same disease as me.					<0.001
Agree	422 (41.0)	105 (60.7)	291 (40.6)	26 (18.6)	
Disagree	607 (59.0)	68 (39.3)	425 (59.4)	114 (81.4)	
Missing	70	13	32	25	
Social media (e.g., Facebook, Twitter) play an important role in finding information, medical specialists or people who have (had) the same disease.					0.072
Agree	257 (24.9)	55 (31.8)	167 (23.5)	35 (23.6)	
Disagree	775 (75.1)	118 (86.2)	544 (76.5)	113 (76.4)	
Missing	67	13	37	17	
I would appreciate patient support groups.					0.004
Agree	341 (32.9)	75 (43.4)	224 (31.2)	42 (28.4)	
Disagree	697 (67.1)	98 (56.6)	493 (68.8)	106 (71.6)	
Missing	61	13	31	17	
I would like to play an active role in the organization of a patient association.					<0.001
Agree	161 (15.5)	53 (30.6)	97 (13.5)	11 (7.4)	
Disagree	878 (84.5)	120 (69.4)	621 (86.5)	137 (92.6)	
Missing	60	13	30	17	
I find it difficult that there is no attention for my disease in the media or in fundraising (charities).					0.592
Agree	295 (28.5)	49 (28.3)	199 (27.8)	47 (32.0)	
Disagree	741 (71.5)	124 (71.7)	517 (72.2)	100 (68.0)	
Missing	63	13	32	18	
In the course of my diagnostic and therapeutic process I have desired a second opinion.					0.037
Agree	293 (28.1)	53 (30.6)	211 (29.3)	29 (19.5)	
Disagree	748 (71.9)	120 (69.4)	508 (70.7)	120 (80.5)	
Missing	58	13	29	16	

^1^ Fisher’s exact (Monte Carlo simulation).

**Table 3:** Satisfaction with care and information needs in rare cancer patients.

Satisfaction with Care and Information Needs	Total *N =* 1099	AYA (18–39) *N =* 186	OA (40–69) *N =* 748	Elderly (≥70) *N =* 165	*p*-Value
How would you rate the care you have received from doctors, nurses, and other health professionals, based on your own experiences?					0.148 ^1^
Bad	6 (0.6)	0 (0.0)	5 (0.7)	1 (0.6)	
Reasonable	62 (5.9)	14 (8.0)	38 (5.2)	10 (6.5)	
Good	261 (24.7)	36 (20.5)	182 (25.1)	43 (27.9)	
Very good	360 (34.1)	65 (36.9)	235 (32.4)	60 (39.0)	
Excellent	367 (34.8)	61 (34.7)	266 (36.6)	40 (26.0)	
Missing	43	9	22	11	
Are you satisfied with the amount of information you received?					0.325
Yes	1011 (96.7)	170 (98.3)	694 (96.1)	147 (97.4)	
No	35 (3.3)	3 (1.7)	28 (3.9)	4 (2.6)	
Missing	53	13	26	14	
Would you have wanted more information?					0.066
Yes	240 (22.9)	39 (22.5)	177 (24.5)	24 (15.8)	
No	807 (77.1)	134 (77.5)	545 (75.5)	128 (84.2)	
Missing	52	13	26	13	
I would have liked more information on the following topics ^2^					0.021 ^1^
**Topics on which More** **Information was Desired**	**Total** ***N =* 280**	**AYA (18–39)** ***N =* 55 (100)**	**OA (40–69)** ***N =* 201 (100)**	**Elderly (≥70)** ***N =* 24 (100)**	
Clarity on diagnosis	10 (3.6)	1 (1.8)	8 (4.0)	1 (4.2)	
**Sarcoma**	**47 (16.8)**	**6 (10.9)**	**34 (16.9)**	**7 (29.2)**	
Sarcoma in general	39 (13.9)	5 (9.1)	28 (13.9)	6 (25.0)	
Rarity of sarcoma	8 (2.9)	1 (1.8)	6 (3.0)	1 (4.2)	
**Treatment**	**73 (26.1)**	**8 (14.7)**	**62 (31.0)**	**3 (12.5)**	
Treatment in general	22 (7.9)	4 (7.3)	18 (9.0)	0 (0.0)	
Surgery	20 (7.1)	2 (3.6)	16 (8.0)	2 (8.3)	
Chemotherapy	3 (1.1)	1 (1.8)	2 (1.0)	0 (0.0)	
Radiotherapy	18 (6.4)	1 (1.8)	16 (8.0)	1 (4.2)	
Alternative treatment	8 (2.9)	0 (0.0)	8 (4.0)	0 (0.0)	
Reconstructive surgery	2 (0.7)	0 (0.0)	2 (1.0)	0 (0.0)	
**Future perspective**	**86 (30.7)**	**23 (41.9)**	**55 (27.4)**	**8 (33.3)**	
Recurrence/survival	42 (15.0)	10 (18.2)	26 (12.9)	6 (25.0)	
Long-term consequences	23 (8.2)	9 (16.4)	14 (7.0)	0 (0.0)	
Survivorship care	21 (7.5)	4 (7.3)	15 (7.5)	2 (8.3)	
**Psychosocial impact**	**10 (3.6)**	**4 (7.3)**	**6 (3.0)**	**0 (0.0)**	
**Practical information**	**8 (2.9)**	**3 (5.5)**	**4 (2.0)**	**1 (4.2)**	
Other	22 (7.9)	5 (9.1)	16 (8.0)	1 (4.2)	
Lifestyle	7 (2.5)	1 (1.8)	5 (2.5)	1 (4.2)	
Fellow patients	6 (2.1)	2 (3.6)	4 (2.0)	0 (0.0)	
Fertility	1 (0.4)	1 (1.8)	0 (0.0)	0 (0.0)	
Form of information	5 (1.8)	1 (1.8)	4 (2.0)	0 (0.0)	
Heredity	2 (0.7)	0 (0.0)	2 (1.0)	0 (0.0)	
Personalized information	1 (0.4)	0 (0.0)	1 (0.5)	0 (0.0)	
**Scientific developments**	**14 (5.0)**	**5 (9.1)**	**8 (4.0)**	**1 (4.2)**	
General comment	10 (3.6)	0 (0.0)	8 (4.0)	2 (8.3)	
Clarity on diagnosis	10 (3.6)	1 (1.8)	8 (4.0)	1 (4.2)	
Would you have wanted less information?					0.774 ^1^
Yes	14 (1.3)	3 (1.7)	10 (1.4)	1 (0.7)	
No	1030 (98.7)	170 (98.3)	710 (98.6)	150 (99.3)	
Missing	55	13	28	14	

^1.^ Fisher’s exact (Monte Carlo simulation). ^2^ Only the categories in bold have been taken into the statistical analysis. AYA: Adolescents and young adults. OA: Older adults.

**Table 4:** Diagnostic and patient intervals and clinical trial participation in rare cancer patients.

Diagnostic and Patient Intervals and Clinical Trial Participation	Total *N* = 1099	AYA (18–39) *N* = 186 (100)	OA (40–69) *N* = 748 (100)	Elderly (≥70) *N* = 165 (100)	*p*-Value
Diagnostic interval					0.001
<1 month	459 (44.3)	67 (37.2)	311 (43.6)	81 (57.0)	
>1 month	576 (55.7)	113 (62.8)	402 (56.4)	61 (43.0)	
Missing	64	6	35	23	
Patient interval					0.001
<1 month	400 (40.7)	54 (32.5)	272 (40.2)	74 (52.9)	
>1 month	582 (59.3)	112 (67.5)	404 (59.8)	66 (47.1)	
Missing	117	20	72	25	
Have treatment options in research context been discussed with you?					
Yes, I have been treated within a trial	92 (8.7)	21 (11.7)	58 (8.0)	13 (8.6)	0.246 ^1^
Yes, however I chose to opt out	33 (3.1)	3 (1.7)	27 (3.7)	3 (2.0)	
No, this has not been discussed	830 (78.4)	139 (77.2)	576 (79.3)	115 (75.7)	
I cannot remember	103 (9.7)	17 (9.4)	65 (9.0)	21 (13.8)	
Missing	41	6	22	13	

^1^ Fisher’s exact (Monte Carlo simulation). AYA: Adolescents and young adults. OA: Older adults.

**Table 5:** Supportive care in rare cancer patients.

Supportive Care	Total *N =* 1099	AYA (18–39) *N =* 186 (100)	OA (40–69) *N =* 748 (100)	Elderly (≥70) *N =* 165 (100)	*p*-Value
In addition to the treatment for your sarcoma, did you receive care from health professionals other than your sarcoma specialists?					<0.001
No	653 (62.1)	86 (48.9)	467 (64.4)	100 (66.7)	
Yes	398 (37.9)	90 (51.1)	258 (35.6)	50 (33.3)	
Missing	48	10	23	15	
**Types of Supportive Care Received**	**Total** ***N =* 899**	**AYA (18–39)** ***N =* 238 (100)**	**OA (40–69)** ***N =* 555 (100)**	**Elderly (≥70)** ***N =* 106 (100)**	***p*-Value**
I received care from the following allied health professional(s)					0.001 ^1^
Psychologist	107 (11.9)	42 (17.6)	63 (11.4)	2 (1.9)	
Sexologist	2 (0.2)	1 (0.4)	1 (0.2)	0 (0.0)	
Social work	43 (4.8)	13 (5.5)	24 (4.3)	6 (5.7)	
Pastoral care	16 (1.8)	4 (1.7)	9 (1.6)	3 (2.8)	
General practitioner	140 (15.6)	29 (12.2)	85 (15.3)	26 (24.5)	
Dietician	47 (5.2)	16 (6.7)	21 (3.8)	10 (9.4)	
Physical therapist	257 (28.6)	66 (27.7)	160 (28.8)	31 (29.2)	
Recovery and balance	41 (4.6)	12 (5.0)	24 (4.3)	5 (4.7)	
Creative therapist	11 (1.2)	4 (1.7)	6 (1.1)	1 (0.9)	
Oncology nurse	66 (7.3)	19 (8.0)	42 (7.6)	5 (4.7)	
Fellow sufferers	23 (2.6)	8 (3.4)	14 (2.5)	1 (0.9)	
Other	146 (16.2)	24 (10.1)	106 (19.1)	16 (15.1)	

^1^ Fisher’s exact (Monte Carlo simulation). AYA: Adolescents and young adults. OA: Older adults.

## Data Availability

The data presented in this study are available on request from the PROFILES registry. The raw data are not publicly available due to privacy.
